# Era of Genomic Medicine: A Narrative Review on CRISPR Technology as a Potential Therapeutic Tool for Human Diseases

**DOI:** 10.1155/2019/1369682

**Published:** 2019-10-07

**Authors:** Odatha W. Kotagama, Chanika D. Jayasinghe, Thelma Abeysinghe

**Affiliations:** ^1^Department of Chemistry, Faculty of Natural Sciences, The Open University of Sri Lanka, Nawala, Nugegoda, Sri Lanka; ^2^Department of Zoology, Faculty of Natural Sciences, The Open University of Sri Lanka, Nawala, Nugegoda, Sri Lanka

## Abstract

Clustered Regularly Interspaced Short Palindromic Repeats (CRISPR) provides acquired immunity in microorganisms against exogenous DNA that may hinder the survival of the organism. Pioneering work by Doudna and Charpentier in 2012 resulted in the creation of the CRISPR/Cas9 genome editing tool on the basis of this concept. The aim of this was to create a rapid, efficient, and versatile genome-editing tool to facilitate genetic manipulation. The mechanism relies on two components: the RNA guide which acts as a sentinel and a Cas protein complex which functions as a highly precise molecular knife. The guide RNA can be modified to match a DNA sequence of interest in the cell and accordingly be used to rectify mutations that may otherwise cause disease. Within a few years following the development of the CRISPR/Cas9 tool, its usage has become ubiquitous. Its influence extends into many fields of biological sciences from biotechnology and biochemistry to molecular biology and biomedical sciences. The following review aims at shedding some light on to the applications of the CRISPR/Cas9 tool in the field of biomedical sciences, particularly gene therapy. An insight with relation to a few of the many diseases that are being tackled with the aid of the CRISPR/Cas9 mechanism and the trends, successes, and challenges of this application as a gene therapy are discussed in this review.

## 1. Introduction

Understanding the genetic basis of human diseases has allowed for substantial progress in biomedical research. Completion of the Human Genome Project and DNA sequence data obtained from diseased individuals have provided an unprecedented opportunity for understanding genetic components allied with human diseases [1]. Alterations in over 3000 human genes are known to be associated with diseases [2]. Monogenic disorders, such as Huntington's disease, cystic fibrosis, thalassemia, and sickle cell anemia, are caused by single-gene mutations while multifactorial diseases such as cancer and diabetes resulted from an interplay between numerous genetic mutations and environmental conditions [3]. Unfortunately, a majority of diseases lack effective treatment strategies; hence, genomic medicine offers a vast potential as an effective therapeutic strategy to combat human disease [1].

Genomic medicine is at the forefront of clinical practice, and it involves rectification of a specific genetic mutation by gene therapy [4]. Gene therapy broadly includes the replacement of a defective gene or genes by an exogenous DNA and editing the mutated gene at its native location [4]. Despite its apparent ease, the introduction of exogenous DNA is associated with a multitude of drawbacks, and complications can be found to be associated with the process. Induction of off-target mutations and erratic effects caused by introduced genes represent a few of such implicated drawbacks. Moreover, its application is limited to a few genetic disorders [4]. On the flip side, however, gene editing elicits a whole new frontier on improving human health. As techniques improve to attempt to make precise, targeted modifications to genome sequences, genetic medicine proves to have extensive promise as a therapeutic intervention against human diseases [4].

The fundamental basis of gene editing lies in the endogenous cellular repair machinery that follows induced DNA double-strand breaks (DSBs) [4, 5]. Breaks in DNA are classically repaired through one of the two major pathways: homology-directed repair (HDR) or nonhomologous end joining (NHEJ). When implementing any of these gene-editing methods, most critical is the precise introduction of a targeted DSB. Currently, four major platforms are in use to induce site-specific DSBs: engineered meganucleases, zinc-finger nucleases (ZFNs), transcription activator-like effector nucleases (TALENs), and most recently the CRISPR/Cas protein system [4, 5]. These techniques have enabled targeted genetic modifications in cultured cells, as well as in animals and plants with high precision.

Compared to other genome editing platforms, CRISPR/Cas9 stands out as being relatively simple as it does not require the engineering of novel proteins for each DNA target site [5]. In CRISPR/Cas9, accurate site-specific changes are mediated by programmable RNA and a restriction enzyme complex referred to as Cas9 gives rise to a highly efficient gene-editing tool [6].

Over the years, this system has been applied in biomedical research, aiming at developing therapeutic interventions for monogenic as well as multifactorial diseases [4]. Thus far, CRISPR/Cas9 technology has been applied for creating animal models for research to mimic diseases or to study disease development by mutating or silencing genes [7]. However, recently its application was extended for editing genes of human embryos as well. The groundbreaking discovery of the ability to repair a mutation in the octamer-binding transcription factor 4 (*OCT4* gene), a gene involved in the development of the human placenta of a human embryo using CRISPR/Cas9, implies a huge clinical potential of treating human genetic disorders [8].

This current review describes advances that entail the use of CRISPR/Cas9 as a therapeutic tool for human diseases. Initially, we discuss the mechanisms of CRISPR/Cas9 protein as a genomic editing tool and then summarize its applications in gene therapy focusing on monogenic diseases such as cystic fibrosis, hemophilia, thalassemia, etc., and multifactorial diseases such as cancers, diabetes, and cardiovascular diseases. Though, CRISPR is identified as the most effective gene-editing tool up to date, much remains to be considered about the reproducibility and ethical issues, particularly with regard to editing the human germline [8]. The occurrence of off-target cleavage is the major drawback of this system and might confound the use of CRISPR/Cas9 in therapeutic applications to some extent. In this light, this review further highlights the current challenges and prospects of CRISPR/Cas9 in genomic medicine.

## 2. The CRISPR/Cas Protein Technology

The CRISPR/Cas protein system was first discovered in 1987 in *Escherichia coli* [9]. However, the acronym was developed in 2002 [6], and it was only in 2007 that it was found that the mechanism is involved in immunological mechanisms of microbes, specifically, prokaryotic microorganisms [10]. The CRISPR/Cas protein immune system was seen to be a novel and highly precise mechanism permitting acquired immunity in bacteria and archaea [11]. Extensive work was subsequently put into pioneering a technique of genome editing that was rapid and precise by Doudna and Charpentier using these findings which resulted in the creation of the CRISPR/Cas9 tool [6].

The CRISPR/Cas system in microbes is represented by a gene locus that provides acquired immunity against viruses and plasmids by targeting nucleic acids in a sequence-specific manner. Such measures allowed for the creation of highly accurate immunological barriers against infection in the cell. It is akin to the cell having a memory of its prior infections [12].

In the given instance that a microbe is infected by an exogenous DNA-bearing entity, the microbe will incorporate segments of the exogenous DNA of the once subdued entity into its own DNA in the form of noncontiguous direct repeats separated by stretches of variable sequences that are known as spacers [13].

When this microbe is subsequently infected by a virus or plasmid bearing a similar genome sequence as the previous entity, the CRISPR sequence helps in generating a complementary RNA (cRNA) strand, making use of the already incorporated palindromic sequences. The cRNA that is accordingly developed has two components to it: the crRNA (CRISPR RNA) and the trans-activating CRISPR RNA (tracrRNA) [9]. Together they generate a highly specific splicing mechanism. The tracrRNA helps mature the crRNA, and the crRNA helps guide the CRISPR-associated (Cas) protein to the target site with the complementary DNA strand. This RNA complex will then act as a guide to the Cas protein complex which will then splice the DNA molecule at the specific site, preventing exogenous DNA from incorporating itself into the genome of the microbe. The generation of a blunt double-stranded break (DSB) at the site of the exogenous DNA helps achieve this. Once the DSB is generated, the cell will direct the DNA to repair itself through one of the two paths to prevent further damage of the exposed DNA molecule [9].

At this point, the cellular mechanism will direct the cell to proceed with nonhomologous end joining (NHEJ) of fragments or with homology-directed repair (HDR). While the former is a rapid and simple process essentially allowing the two separated components of the DNA molecule to join together, it is inefficient and can generate errors by frameshift mutations [14]. Accordingly, in most instances, the cell directs its mechanisms to carry out homology-directed repair (HDR). This process is of higher precision and involves the use of a homologue's DNA template. Given the diploid nature of cells, this process can be achieved easily in the cell. This basic process is that which has been refined and artificially induced in living cells to generate the CRISPR/Cas9 system [15].

A third less frequented mechanism for repairing DSB lies in what is referred to as microhomology-mediated base paring. This type of repair is found to be associated with alternate NHEJ which does not rely on Ku proteins and recombination factors (like classical NHEJ). Despite it being highly error prone, many studies have used this method to generate less labour-intensive processors in germ line modification [17]. Furthermore, microhomology-mediated base pairing together with CRISPR has also been developed to form an efficient tagging tool that will greatly aid functional analysis of proteins in their native state [18].

## 3. Common Genomic Editing Approaches

### 3.1. Zinc-Finger Nucleases (ZFNs)

Zinc-finger nucleases (ZFNs) are a versatile and effective gene-editing approach, which has separate DNA-binding and DNA-cleaving domains [19]. The DNA-binding domain consists of eukaryotic transcription factors and zinc finger(s). The DNA-cleavage domain contains FokI restriction enzymes, and it is responsible for the catalytic cleavage of target DNA [19]. These modified complexes can then be induced to bring about a DBS in the genome of a cell by cleaving a specific genome sequence. As the ZNF can only recognize a restricted number of bases, site-specific cleavage of the genome is induced by manipulating the ZFN complex to recognize two sequences that are found on either side of the target site [19]. Following the identification of the relevant site, cleavage of the genome will take place mediated by the FokI restriction enzyme, thereby generating a DSB in the genome which can then be manipulated as required [19].

### 3.2. Transcription Activator-Like Effector Nucleases (TALENs)

These function in a manner similar to ZFNs but are of different origin. TALENs refer to a group of proteins that are developed by plant pathogenic bacteria to aid in their process of infection [13]. The proteins that are developed by these bacteria have a sequence of about 33 to 35 amino acids. Within this, it is common to come across between 13 and 28 repeats. Polymorphism among the results of the repeat are used in the development of repeat-variable diresidues (RVDs). These RVDs have a high level of nucleotide preference and therefore can be used to build proteins that can recognize genome sequences from base to base [20]. Similar to the use of ZFNs indicated above, these protein structures can be led to bind with a FoKI restriction enzyme and bring about a DSB in the genome.

While both techniques are relatively well developed, they are quite costly and time-consuming, requiring many specific proteins to be modeled based on the requirement, as a whole resulting in a comparatively inefficient gene-editing tool [13].

### 3.3. RNA-Guided Engineered Nucleases (RGENs)

RNA guided engineered nucleases represent a tool for genomic editing that is, unlike ZFNs and TALENs, derived and applicable to the CRISPR/Cas9 mechanism. Fundamentally, a similar role is played by these engineered nucleases as the typical CRISPR/Cas9 protein complex. The nucleases will generate double-strand breaks (DSBs) at certain specific, chosen points. Controlled mutations can then be generated in order to repair the induced DSBs. However, initial work with this type of complex indicated a high incidence of unwanted DNA integration derived from the Cas9 plasmid and the synthetic RNA as well [21]. This condition could be potentially hazardous for therapeutic applications. Hence, recently attempts were taken to improve the precision of these nucleases [22]. The specificity was increased by introducing mutant Cas9 proteins (enhanced specificity Cas9 (eSpCas9) and Cas9-high fidelity (Cas9-HF)) [22]. With these proteins, it was anticipated to reduce the nonspecific interactions between a Cas9-RNA complex and its substrate DNA that subsequently would reduce off-target activity [22]. However, these systems were reported to be poorly active at some target sites [22, 23]. Hence, another approach with a hairpin secondary structure engineered onto the spacer region of single guide RNAs (hp-sgRNAs) has shown increased the specificity of various CRISPR effectors [23].

## 4. Application of CRISPR/Cas9 as a Therapeutic Tool for Human Diseases

Two of the major aspects in the biomedical field to which the CRISPR/Cas9 protein tool kit has been introduced to are in the diagnosis and treatment of genetically linked diseases [24]. The degree of development of the CRISPR/Cas tool is extensive. A vast amount of research has been carried out with respect to this, and the potential to extend beyond animal models that are now being made use of has been capaciously highlighted [25].

Furthermore, the use of the genome-editing tool extends beyond genome editing itself. Chen et al. [26] showed how the system could be used for the purpose of visualizing the gene and its associated components. Their work revealed far better understanding with relations to the workings of telomeres and subnuclear localization of the membrane mucin (*MUC4*) gene loci. Insight into the cohesive nature of the *MUC4* loci on sister chromatids during replication along with their dynamic behavior during mitotic cell division was also revealed [26]. However, this review mainly focuses on the application of CRISPR technology as a therapeutic tool for many diseases. The application of CRISPR technology for monogenetic disorders and multifactorial diseases will be dealt with separately in this review.

### 4.1. Monogenic Disorders

Monogenic disorders arise from a defect in a single gene and are inherited according to traditional Mendelian patterns [27]. These disorders affect millions of people, and it has been estimated that over 10,000 human diseases fall under this category. Monogenic disorders are mainly classified as dominant, recessive, and X-linked [27]. Treatment for most of these diseases remains to be managing symptoms and focusing on disease management without addressing the underlying genetic defect. The advent of gene-editing tools shows great therapeutic promise against monogenetic disorders [5]. Herein, we briefly describe the potential of preclinical CRISPR-Cas9-based therapeutic interventions (Table 1).

### 4.2. Cystic Fibrosis

Cystic fibrosis (CF) is an autosomal recessive genetic disorder characterized by secretion of abnormal amounts of a viscous fluid in the airways of the lungs and in the ducts of the pancreas [47]. Though CF is a multiple organ disorder, morbidity and mortality are mostly associated with airway or lung obstruction [48].

CF is caused by mutations in the cystic fibrosis transmembrane regulator (*CFTR*) gene [49]. The CFTR protein functions as an anion channel, which is regulated by protein kinase A-dependent phosphorylation at the cell membrane; mutations disrupt the electrolyte homeostasis of epithelial [50]. The homozygous deletion of F508 (CFTR F508 del) results in a deletion of phenylalanine at position 508 (DF508) in nucleotide-binding domains (NBD1) that subsequently affects the protein folding, plasma membrane expression, function, and stability of CFTR [48].

CRISPR/Ca9-mediated homologous recombination has successfully corrected the mutant F508 del in intestinal stem cells isolated from CF patients and demonstrated the functionality of the corrected allele in the organoid system. Patients' organoids (small and large intestine) were independently transfected with single-guide RNAs (sgRNAs) targeting either CFTR exon 11 or intron 11, together with a donor plasmid encoding wild-type CFTR sequences. The successful correction was recorded and could rescue the CFTR phenotype in organoids. This was utilized as a proof of principle study to implicate the safety of the clinical study [28].

Though this is an effective strategy, these cells cannot be utilized to study the pathophysiology of CF in the lung. In a parallel study, a customized CRISPR system was used to correct the homozygous deletion of F508 in the *CFTR* gene in induced pluripotent stem cells (iPSCs) generated from CF patients. The CRISPR system consisted of a plasmid encoding the full-length Cas9 protein for optimal expression in human cells and was driven by the stem cell-compatible eukaryotic transcription elongation factor 1 alpha 1 (*EEF1A1*) promoter and a separate plasmid containing a U6 promoter-driven gRNA hairpin cassette. It was observed that the corrected iPSCs expressed normal CFTR expression and function when they differentiated into mature airway epithelial cells [29]. Hence, it is suggested CRISPR technology has a promise in treating CF. Particularly, the isogenic iPSC model may provide an optimal approach when considering clinical applications.

### 4.3. Sickle Cell Anemia

Sickle cell anemia (SCA) is a severe monogenic disorder that results in abnormal sickle-shaped erythrocytes [51]. These sickled cells are deprived of oxygen-carrying capacity, and the overall functionality of the red blood cells is impaired (RBCs) [51]. SCA is caused by a point mutation in the *β*-globin gene (*HBB*) [31] which is characterized by sickle-shaped hemoglobin (HbS). A single nucleotide substitution from A to T in the codon for the sixth amino acid replaces glutamic acid by valine, and this alteration causes the production of HbS [31]. Thus, genome editing has been implicated in potential therapy.

Application of CRISPR/Cas9 technology as a genome-editing technique has resulted in 18% gene modification in blood-derived CD34 + cells obtained from SCA patients under *in vitro* conditions. Interestingly, CRISPR/Cas9 also corrected bone marrow-derived, CD34 + hematopoietic stem and progenitor cells from sickle cell disease patients, leading to the production of wild-type hemoglobin. It is interesting to note that CRISPR systems had a higher average of *β*-globin gene disruption compared to TALENs which were utilized to compare the efficiency of the gene correction by the two genome-modifying tools [30].

Park et al. [31] used an optimized CRISPR/Cas9 system and a donor template to achieve nearly 30% HDR rates in CD34^+^ cells. 80% of the cell were viable and persisted in the population over the course of differentiation while retaining the potential for differentiation into erythroid cells. Furthermore, these cells were able to produce wild-type *β*-globin. Thus, the efficacy of correction can be increased with the optimization of the CRISPR technique. The CRISPR has also been used to correct the Glu6Val mutation responsible for sickle cell disease by using patient-derived stem and progenitor cells. This homologous recombination at the *HBB* predicts novel therapies for *β*-hemoglobinopathies [32].

### 4.4. Thalassemia

*β*-Thalassemia is a hematological disorder characterized by reduction or absence in the synthesis of hemoglobin (HB) subunit *β* (HB *β* chain) and is among the most common genetic disorders worldwide [52]. Thalassemia may be caused by more than 200 different point mutations and, rarely, deletions in the *HBB* gene [52].

The IVS2-654(C > T) is a mutation that is common in Southeast Asia [53]. A site-specific correction has been achieved in patient-derived iPSC using the CRISP/Cas9 system [33]. Similarly, CRISPR/Cas9 has applied to correct the *HBB* gene CD41/42 mutation in *β*-Thal iPSCs. The cells have exhibited normal karyotype and have retained full pluripotency, and it was revealed through whole exome sequencing that the mutation load to the exome was minimal [34].

CRISPR/Cas9 has been employed to correct mutations in human naïve iPSCs obtained from urinary cells of *β* thalassemic patients. The naïve iPSCs have exhibited marked gene correction efficiencies compared with that of primed iPSCs. These cells were capable of hematopoietic differentiation and thus provided an excellent source for further clinical application [35].

CRISPR/Cas9 technology with the combination of the *piggyBac* transposon has efficiently corrected the *HBB* mutations in iPSCs obtained from thalassemia patients. The correction has been highly successful without any off-target effects, and cells have exhibited normal karyotypes with full pluripotency [36].

CRISPR/Cas9 technology has also been used to correct *β*-Thal iPSCs cells and develop improved hematopoietic differentiating ability [37]. Corrected *β*-Thal cells have exhibited normal karyotypes and full pluripotency as human embryonic stem cells (hESCs) showed no off-target effects with restored HBB expression compared to unresolved controls [37].

### 4.5. Huntington's Disease

Huntington's disease (HD) is an autosomal-dominant, progressive neurodegenerative disorder with a distinct phenotype, including chorea, dystonia, incoordination, cognitive decline, and behavioral difficulties [54]. HD is characterized by expansion of a CAG repeat in the huntingtin gene (*HTT*) that results in an elongated polyglutamine tract in the huntingtin protein [55]. Recent research indicates the capacity to make use of the genetic toolkit to silence or neutralize the mutant HTT gene responsible for causing the disease. Personalized CRISPR/Cas9 system was used in order to selectively silence only mutated gene responsible for HD, implying the potential for a person with a dominant allele linked to a genetic disease to be resolved in a similar manner. The personalized system generated relied on dual gRNA PAM (protospacer adjacent motif)-altering SNP-based allele-specific CRISPR/Cas9 in order to bring about knockout of mutations. The use of PAM-altering variants allowed for precise selectivity, preventing the transcription of mutant HTT mRNA. The wide variety of haplotypes associated with HD indicates the strong need for such a personalized system [38].

Research was carried out in order to isolate and alter the expression of the HD gene with positive outcomes [39]. CRISPR/Cas9 technology was used for allele-specific correction with the advantage of highly prevalent single-nucleotide polymorphisms (SNPs) in the *HTT* locus, and it was evident the reduction in the expression of mutant *HTT* alleles in human HD fibroblasts [39]. The allele-specific HTT exon-1 deletion was achieved using a SNP (single-nucleotide polymorphism)-dependent PAM in the HTT promoter in combination with a common guide in intron 1 and resulted in an elimination of N-terminal and C-terminal protein fragments. Moreover, the sgRNA/Cas9 complexes were effective in *in vivo* HD mouse model [39].

It was observed that the loss of the *HTT* gene in mice can lead to embryonic lethality, and CRISPR/Cas9-mediated inactivation effectively depleted HTT aggregates and attenuated early neuropathology. The reduction of mHTT expression in striatal neuronal cells in adult HD140Q-knocking mice did not affect viability but alleviated motor deficits [40].

In another study, in order to prevent the production of mHTT protein, two CRISPR/Cas9 plasmids were created. One was used to cut the DNA at the untranslated region upstream to the uORF (open reading frame) and the other to cut the DNA at the exon-1-intron boundary. Results were seen to negatively influence the translation of mHTT protein [56].

### 4.6. Duchenne Muscular Dystrophy

Duchenne muscular dystrophy (DMD) is a lethal X-linked recessive muscle-degenerative disease [57]. It is caused by a mutation in the dystrophin gene [38]. CRISPR/Cas9 has been used for exon knocking of the dystrophin gene of patient-derived induced pluripotent stem cells (iPSCs) successfully [41]. The multiplex gene-editing capability of the CRISPR/Cas9 system facilitates the generation of a single large deletion that can correct up to 62% of DMD mutations. CRISPR was used to target exons 45–55 and introduced shifts within exons or brought about deletion in one or more exons allowing for the normal phenotype in human myoblasts [41]. Exon knocking using CRISPR was found to be effective in restoring the dystrophin protein in patient-derived iPSCs. The expression of full-length dystrophin protein was observed when iPSCs were differentiated toward skeletal muscle cells [42].

### 4.7. Hemophilia A

Hemophilia is an X-linked bleeding disorder with two main forms: hemophilia A and hemophilia B. Hemophilia A and hemophilia B are manifested with deficiency of clotting factor VIII and IX, respectively [58]. Most severe hemophilia A cases have resulted from two gross (140-kbp or 600-kbp) chromosomal inversions that involve introns 1 and 22 of the *F8* gene, respectively [43]. The CRISPR technology has corrected the mutation in the F8 gene and has revered the chromosomal segments back to the WT situation in iPSCs derived from patients [43]. Hemophilia B is frequently manifested by the Y371S mutation. A novel mutation, Y371D, in the human F9 gene which attributes to the severe form of hemophilia B has also been corrected by CRISPR/Cas9 modified mouse models and proposed as potential therapeutic interventions [44].

### 4.8. Chronic Granulomatous Diseases

Chronic granulomatous disease (CGD) is a commonly encountered immunodeficient condition associated with defective phagocytes characterized by the inability to produce NADPH oxidase and reactive oxygen species (ROS) [59]. Consequentially, phagocytic cells lose the ability to destroy pathogenic microbes such as bacteria and fungi because of which infection and recurrent inflammation are responsible for potentially life-threatening situations. Inflammation of hallowed viscera such as the gastrointestinal tract and the genitourinary track represent the most common clinical manifestations while infections of the lungs, retina, bones, and a host of other vital organs have been observed owing to the contagious and hematogenous nature of infections [60].

CGD has its etiologies, genetically, associated with two main forms. One being an X-linked chromosomal mutation associated with the gp91^phox^ gene commonly referred to as X-linked granulomatous disease which is responsible for 65%–70% of clinical cases in the United States [61] and the other known as autosomal chronic granulomatous disease manifesting owing to an array of mutations associated with p22phox, p40phox, p47phox, and p67phox genes [62].

Despite the ability to mediate disease progression and symptomatically treat infections using a combination of broad-spectrum antibiotics or steroids, relapses are often observed especially with an association to patients suffering from complete or extensive NADPH oxidase impairment [62]. Accordingly, considerable attention has been given to gene therapy as an alternative treatment pattern to develop more durable outcomes. An extensive review by Keller et al. [63] delves into the attempts that have been carried out to mediate CGD with the aid of gene therapy throughout the years.

In this regard, a large amount of works have been done with the use of ZFNs and TALENs with success being achieved through each mechanism, and scientists have now begun to turn their attention to the use of CRISPR/Cas9 tool as well. In 2015, Flynn et al. [45] began to delve into the use of the CRISPR/Cas protein mechanism by attempting to promote rectification of endogenous genes through homologous repair. Successful gene editing was achieved, and restoration of the oxidative capacities was observed in iPS-derived phagocytes extracted from a CGD patient suffering from an intronic mutation in the CYBB gene [45]. More recently, promising work was carried out by De Ravin et al. [46], and it was shown that the CRISPR/Cas9 protein gene-editing tool was used to correct mutations in blood stem cells of patients suffering from CGD. 31% of cells exhibited gp91^phox^ expression, and when corrected cells were engrafted into mice, stable expression of the gp91^phox^ gene was observed for five months [45].

## 5. Multifactorial Diseases

In contrast to monogenic diseases discussed above, multifactorial diseases stem through far more complicated processes. Cancer, diabetes, and cardiovascular diseases, with which we will be dealing with in this review, are commonly encountered multifactorial diseases responsible for an extensive number of human deaths worldwide. As put by Todd [64], disease occurrence is attributable to the interaction with the environment of alleles at many loci interspersed throughout the genome. Owing to this complex nature, extensive research has been carried out to counter these diseases and attempt to find preventive solutions. As such, it is of no surprise that CRISPR technology has successfully been capable of addressing many queries in this regard.  Table 2 summarizes the application of CRISPR technology as therapeutic tools for multifactorial diseases.

### 5.1. Cancer

Cancer involves multiple genetic alterations, unlike monogenic disorders. The onset of cancer typically involves a series of mutations in the genome resulting in uncontrolled cell proliferation, lack of apoptosis, and alterations in epigenetic regulation [79]. The CRISPR/cas9 system is able to edit multiple genes in parallel and directly target the causes of cancer, and experimentation has been seen to be promising on many accounts [80]. The potential for the use of the CRISPR tool in the treatment of cancer grew exponentially after the discovery that the genome-editing tool could be used to manipulate and induce cancer-generating mutations as well. The breakthrough began with Hu et al. [65] who studied high-risk human papillomavirus (HR-HPV) which has been shown to be a major causative agent associated with human cervical cancer. HPV infection is seen to cause mutations associated with E6 and E7 genes which play vital roles in maintaining the malignant nature of cancer. It was revealed that using a modified CRISPR/Cas9 system (HPV16-E7 gRNA guided CRISPR/Cas system), which disrupts HPV16-E7 DNA at specific sites, would result in apoptosis and growth inhibition of HPV-positive SiHa and Caski cells, but not in HPV-negative C33A and HEK293 cells. It was further observed that the disruption of E7 DNA directly led to the downregulation of E7 protein and upregulation of tumor suppressor protein pRb [65].

Further developments came about with the use of the CRISPR in modified forms to induce tumorigenesis in model animals in a much simpler and convenient manner allowing for the ability to better study cancer and develop cancer models. Direct mutation of cancer generating genes was carried out by Xue et al. [66] in the liver cells of mice targeting Pten and p53 genes. When these mutations were induced simultaneously, it was seen that liver tumors were generated mimicking those caused by Cre-loxP-mediated deletion of Pten and p53. In the instance that the Pten gene was mutated alone, elevated Akt phosphorylation and lipid accumulation in hepatocytes were identified; an outcome phenocopying the effects of deletion of the gene using Cre-loxP technology [66]. Similar studies were carried out extensively leading to the uncovering of many underlying processors associated with cancer and its development [81]. The technology has been extended and developed to carry out more than just generating animal models in association with cancer studies.

In myeloid malignancies, somatic mutations of the epigenetic modifier and tumor suppressor ASXL1 are common. In a study carried out by scientists with the aid of xenografted mice in which mutations were corrected in myelogenous leukemia cell line (KBM5), cell lines showed significantly longer survival in relation to those xenografted with uncorrected cell lines. Here, the CRISPR/Cas9 tool was utilized to rectify the ASLX1 homozygous nonsense mutation present in KBM5 cell lines. The rectification restored normal cellular function and downregulated polycomb repressive complex 2 (PCR2) target genes [67]. Likewise, successful use of the CRISPR/Cas9 mechanisms was seen in efficiently silencing the cyclin-dependent kinase 11 (CDK11). CDK11 is a gene vital for the proliferation of osteosarcoma cells. Signaling by CDK11 is found to be essential in cell growth and proliferation, and silencing of the CDK11 gene was seen to be associated with decreased cell viability and proliferation and also seen to induce apoptosis in KHOS and U 2O cell lines. The knockout of the gene was also seen to be associated with reduced invasion and migration of cells [68].

Further studies carried out in this effect have shed light on a vast amount of information, especially with regard to the use of viral vectors carrying drug-inducible sgRNA to elicit Cas-9-mediated mutations in the gene of interest. Studies have shown the broad applicability of this system in knocking out mutations as well as their induction with ease. For example, myeloid leukemia cell differentiation protein (MCL1), an antiapoptotic protein, is essential for the sustained survival of human Burkitt lymphoma (BL) cells. Studies by Aubrey et al. [69] have shown the capability of inhibiting MCL-1 in human BL cells by using a lentiviral CRISPR-Cas9 platform, which resulted in the apoptosis of BL cells at a very high frequency. Moreover, in human BL xenograft models *in vivo*, dramatic tumor regression or impaired growth by repeated induction of sgRNA was observed [69].

CRISPR-Cas9 mediated knockout of particular genes such as SHCBP1 (SH2-domain binding protein 1) in breast cancer cells *in vitro* [70] and KLHDC4 (kelch domain containing 4) in nasopharyngeal carcinoma cells, both *in vitro* and *in vivo* [71], has shown to generate positive outcomes, with both knockouts associated with reduced proliferation of cells as well as inhibited cell migration and invasion in the latter.

The gene-editing tool kit has also been successfully modified on many accounts to generate drug resistance. For example, the use of the CRISPR-Cas9 system to target the ATP-binding cassette subfamily B member 1 (ABCB1) gene in MDR (multidrug resistant) cell lines used to block the expression of P-gp (P-glycoprotein 1) which is associated with poor patient survival. Successful reversal of resistance to doxorubicin was shown via analysis [82].

Presently, the use of CRISPR/Cas9 mechanisms has been extended to study compilations of CRISPR cancer vulnerability screens and drug target screens. The onset of many cancers has been known to be owing to oncogenic virus, such as human papillomaviruses (HPV) in cervical cancer (as shown in this review), Epstein-Barr virus (EBV) in nasopharyngeal carcinoma, and hepatitis B virus (HBV) and hepatitis C virus (HCV) in liver cancer. Shi et al. [83] have screened 192 chromatin regulatory domains in murine acute myeloid leukemia cells targeting CRISPR-induced mutations to the 5′ exons of candidate genes. Six known drug targets and 19 additional dependencies have been identified in this study [83]. Therefore, the ability to inactivate or eliminate these pathogens, which can interrupt or even reverse tumorigenesis, represents a promising anticancer strategy for patients with virus-associated cancers.

### 5.2. Diabetes

Diabetes is a common multifactorial disease that manifests in individuals of all ages and presently is a major blow on global human health. The WHO in 2017 put forward figures confirming these impending fears. It has been shown that the number of persons suffering from diabetes has grown from 108 million in 1980 to 422 million by 2014 with a global prevalence of the disease among young adults over the age of 18 rising to 8.5% [84]. It is also predicted that diabetes will be the seventh leading cause of death by 2030 [85].

Etiologies of diabetes are highly variable. Two major forms of diabetes exist, commonly referred to as Type 1 and Type 2 [71]. The former (Type 1) commonly referred to as insulin-dependent diabetes is seen to be associated with an increase or decrease in the frequency of specific histocompatibility antigens on the 6^th^ chromosome and with islet cell antibodies. Although more fondly referred to as juvenile diabetes, this is no longer considered accurate as it may occur at any age. The latter (Type 2) is the most prevalent form of diabetes that is referred to as noninsulin-dependent diabetes and primarily owing to obesity and unhealthy lifestyles among individuals. While insulin production is present in this form, the quantity produced may be insufficient to meet the body's needs or in some cases may be owing to the development of insulin resistance [86].

As with most developments in relation to understanding diseases, the breakthrough on the application of this field in combating diabetes comes about with the development of animal models to better understand the pathways and physiologies of the disease. Successful generation of insulin-deficient piglets by the disruption of the INS gene via CRISPS/Cas9 [72] was achieved by a group of scientists possibly paving the way for developments in the use of pig models in studies. Concurrent studies show the development of mouse models where co-microinjection of sgRNA and Cas9 mRNA helped in the generation of *Letine gene* and *Leptin receptor gene* knockout mice [73]. These mice showed identical phenotypic outcomes to mice models involving the use of obese and diabetic mice [73].

The use of CRISPR has been seen to set the pathway to generating animal models in a relatively convenient manner. The development of models for diabetes has been extended to human stem cells via the use of CRISPR/Cas9. Human pluripotent cells that are capable of differentiating into any type of cell and are self-renewing have been mutated through the use of the CRISPR/Cas9 tool. Here, mutations were induced in the hepatocyte nuclear factor (HNF) 1b gene that is thought to know to cause diabetes via pancreatic hypoplasia and *β*-cell dysfunction [74].

Furthermore, skin grafts from mouse and human epidermal progenitors were engineered by using the CRISPR technique to controllably release GLP-1 (glucagon-like peptide 1), which regulates blood glucose homeostasis. Induction of GLP-1 induction onto immunocompetent hosts showed increased insulin secretion, and insulin resistance indicates a clinical potential of developing a safe therapeutic approach [87].

### 5.3. Cardiovascular Disease

Cardiovascular disease (CVD) remains to be one of the greatest causes of morbidity and mortality worldwide. Recent statistics indicate that CVD is responsible for 31% of deaths globally. In the United States, in 2017 alone 800,000 deaths were caused by CVD; to put it in perspective, it accounts for 1 in every 3 deaths [88].

Integrating genomic medication in the arsenal for the fight against CVD is imperative. Genetic editing and the use of the CRISPR/cas9 gene editing tool have allowed for a large amount of development. In this regard, the diseases were attempted to be rectified via genomic therapy, and the formulation of animal models plays a vital role. The application of the CRISPR/Cas9 mechanism has been shown to be successful in the generation of mouse models to better understand disease etiologies associated with CVD. Recent work published [89, 90] includes a comprehensive review that brings together various aspects of the application of the CRISPR/Cas protein genomic tool in cardiovascular research along with its limitations. Studies have been extended to include zebrafish as model animals as well. Scientists successfully made use of the CRISPR/Cas9 mechanism to modify zebrafish embryos with a knockout of a *LMNA* gene which is a gene homologous to the human gene *LMNA.* The latter is found to be responsible for early onset cardiac conduction death [75].

Similarly, the requirement for pig models in the study of CVD, which are highly sought after in the fields of biomedical research owing to extensive similarities with humans, was bridged with the creation of six biallelic knockout pigs. Apolipoprotein E (*ApoE*) and low-density lipoprotein receptor (*LDLR*) genes were simultaneously targeted with the use of the CRISPR/Cas9 system, and the knockout models were generated in a single step, thus creating an ideal model for the study of CVD [78].

Studies have also been carried out in order to attempt rectification and modification of mutations associated with a high incidence of CVD. In one study in mice, *PCSK9* (proprotein convertase subtilisin/kexin type 9) gene responsible for generating increased levels of PCSK9 in the blood which in turn causes elevation of low-density lipoprotein cholesterol (LDL-C) by acting antagonistically on LDL receptors was seen to be successfully mutated to form loss of function types. The outcomes in the study were very successful with >50% of mice showing loss of function in the PCSK9 gene and in turn reduced levels of LDL. This was found to reduce plasma glucose levels by 30%–45%. What is greatly noteworthy was that the degree of off-target mutations was virtually absent in the 10 selected sites [76]. A more recent study in February 2018 made use of a modified version of the CRISPR/Cas mechanism known as Base Editor 3 (BE3). Base editors can generate changes in the base of the genome, and the particular form used in the study was capable of introducing cytosine to thymine changes at the desired sites. The genome editing tool was used to generate a loss of function angiopoietin-like-3 gene (*ANGPTL3*) which is associated with reduced risk of CHD and reduced blood triglycerides and LDL [77].

## 6. Challenges of Application of CRISPR/Cas9 Gene-Editing Tool

Despite the great promise of the CRISPR-Cas9 technology as a genome-editing system, there are several challenges that should be addressed [91]. Lack of safe and efficient delivery systems, off-target effects, and ethical considerations have been identified as the major barriers of extending CRISPR-Cas9 system in clinical applications [92].

### 6.1. Delivery Systems of CRISPR/Cas9

The biggest challenge thus far with the CRISPR system is the delivery to the target cells [92]. Efficient delivery of the CRISPR/Cas protein tool stands as a major requirement to minimize off target effects within the gene and to ensure that the desired cell or tissue is reached by the tool [91]. Several physical and viral systems have been exploited for CRISPR-Cas9 delivery to target site. The physical system includes the following: electroporation, gold nanoparticles, lipid-mediated transfection, cell penetrating peptide, mechanical cell deformation, hydrodynamic delivery, DNA nanoclews, microinjection, and induced transduction by osmocytosis and propanebetaine [92]. These physical methods are safe to use compared to that of viral vectors, and there is no size limitation for transgenic DNA. Furthermore, the availability and cost effectiveness have increased their applications [92]. Physical delivery of CRISPR-Cas9 is effective in producing knockout cell lines and animal models; however, relatively poor delivery efficacy has been reported in *in vivo* applications [92].

Viral delivery systems have been identified as most efficient systems to deliver plasmid-based nucleic acids to mammalian cells *in vitro/in vivo*. Hence, deliver plasmid-based CRISPR-Cas9 to mammalian cells [92]. There are two types of viral system frequently used in gene transduction: adeno-associated virus (AAV) and lentivirus [93]. The AAVs are nonpathogenic and mild immunogenic and exhibit serotype specificity and ability to infect dividing and nondividing cells. The challenge with the AAV-mediated CRISPR/Cas9 is the packing limitation of AAV [92]. To overcome the problem, attempts have been made with the dual AAVs that can separately deliver Cas-9 encoding DNA and sgRNA [94]. However, injection of two AAVs into one target cell is also challenging. Lentivirus-mediated CRISPR-Cas9 has achieved successful result in both *in vitro* and *in vivo* systems [95]. The biggest advantage of lentivirus is the high infection efficiency even in nondiving cells, and thus it is crucial for gene modification of cells like liver and brain [92].

### 6.2. Off-Target Effects

The low efficiency of HDR is one of the main challenges with CRISPR-Cas9-based therapy [91]. When repairing DSB repair mechanisms, NHEJ is more efficient compared with HDR and is suitable for generating indels to knock out mutations. Considerable progress has been made to increase the efficiency of HDR. Recent developments indicate the use of a modified CRISPR/Cas9 system involving the use of a mutant Cas9 domain to produce CRISPR nickase. The use of this altered system has been found to be more specific and efficient in reducing the number of off-target effects considerably [96].

Perhaps, one of the most complex issues lies in the minimization of off-target effects when utilizing the CRISPR/Cas protein system. Generating modified systems which have far high specificity and more efficient gene targeting has been undertaken by many scientists as evident by the plethora of work being carried out. Especially more so in clinical trials where proper targeting of drugs must be achieved, the absence of completely successful methods of targeted delivery as yet proves to be a major drawback [91].

Thus, studies have been carried out to increase the efficiency and specificity of the CRISPR/Cas protein system involving the prioritization of HDR over NHEJ. Chu et al. [97] showed how ligase IV inhibitor SCR7 or coexpression of adenovirus 4 EB1B55K and E4 or f6 proteins could be used in order to silence KU70, KU80, or DNA Ligase IV, which are key factors allowing NHEJ. Thus, the intensity of HDR was increased by four to five-fold [97].

### 6.3. Ethical Issues

CRISPR/Cas9 system has emerged as a potential therapeutic tool for many diseases. However, there are many ethical considerations involved in the application of this technology into preclinical or clinical trials [98]. The major ethical consideration of CRISPR technology is the concerns about the potential and technical limitations of CRISPR technology. The possibilities of off-target effects, incomplete editing, and limited efficiency constrained the application of CRISPR into clinical applications [98]. Also, it is uncertain whether modified organisms will be affected indefinitely and whether the correction will be inherited [98]. Furthermore, CRISPR application is limited due to the fact that the genetic makeup and the biological phenotypes are not fully understood [98].

Since the inception of the CRISPR tool in 2012, the degree of development the tool has reached is extensive and that too within a relatively short period of time. One of the major drawbacks is that we, yet, do not understand the long-term effects that tampering with the gene pool may have. Given that we do not understand the effects of these gene alterations, we have no knowledge as to how to mediate the adverse impacts that may be generated by these genetic modifications. For example, studies have been carried out to develop a gene drive that could reduce the population of malaria carrier mosquitoes by bringing about female sterility following disruption in AGAP005958, AGAP011377, and AGAP007280 genes via the CRISPR/Cas9 mechanism [99].

Despite the positive basis these studies consolidate, we may be unintentionally laying the foundation required for the development of new epidemics by generating genomes that are more compatible with much more virulent strains. Apart from this, the incorporation of gene drives into living organisms may convert once noninvasive species into highly invasive forms that could wipe out the native species in a matter of decades. It is vital then to understand that despite the immense advantages that are brought about by the CRISPR/Cas9 toolkit, proper understanding and usage of the CRISPR/Cas9 tool must be developed in order to reap the maximum benefit with minimal compromise.

No discussion regarding the ethical implications can be completed without taking into consideration the growing concern regarding the modification of the human germline. Recent developments indicate that a major consideration is to be given with regard to the genome editing in human embryos, with many scientists strongly believing that humanity is still not yet ready to tamper with human embryos.

### 6.4. Emerging CRISPR Technologies

Genome editing with CRISPR/Cas has rapidly gained popularity, and the quest for improving the efficiency of this system continues. Several novel approaches have been introduced to increase the efficiency of the CRISPR system. Base editing, a new CRISPR/Cas-based approach, can precisely convert one nucleotide to another in DNA or RNA without inducing a double-strand DNA break [100]. Developments have also come about with the generation and application of base editors [101], and development of several base editors has been carried out by combining different nucleobase deaminases with Cas9 or Cpf1 proteins. In order to further develop the CRISPR system by reducing the nonspecific interactions between the Cas9 protein and the target DNA, scientists have been working on creating alternate Cas9 proteins. A commentary by Nakade et al. [102] draws attention to the development of mutated forms of Cas9 such as eSpCas9 and SpCas9-HF.

Various modifications and developments have been carried out to further improve this system as indicated in the commentary. New tools based on the CRISPR/Cas9 tool such as CRISPR-Cpf1 includes a number of advantages such as low cost, high efficiency, and a number of factors which could not be obtained with the traditional system. This system which makes use of an endonuclease from type-v CRISPR/Cas9 allows DNA cleavage with a single crRNA, producing cohesive ends with 4 or 5-nt overhangs, and allows for multiplex genome editing and a number of other advantages that are not achieved with CRISPR/Cas9 [103].

## 7. Conclusion

The CRISPR/Cas9 system is a versatile gene-manipulating tool consisting of a guide RNA sequence (sgRNA) and a DNA splicing protein complex. The development of this has allowed inducing DSB at selected sites determined by the modification of the guide RNA as required. An immense potential thus exists with relation to genomic medicine as diseases associated with defective genes can be rectified at the level of the genome itself. Scientists have been attempting to rectify both monogenic as well as multifactorial diseases with the use of CRISPR/Cas9.

Their efforts attempt not only to remedy mutations but also to make use of gene manipulation tools in order to construct animal or human models that will help study the pathology of diseases and their development. The large quantity of information that has been accumulated in this regard has allowed scientists and physicians to fully understand the causes and the extent of many diseases.

Despite the availability of gene-editing tools such as ZNFs, TALENs, and RGENs, CRISPR/Cas9 has facilitated reaching this goal at a much faster pace primarily owing to the ease of use and its high versatility. However, while on this journey of uncovering knowledge, we should be mindful of the limitations that are imposed by the CRISPR/Cas protein tool. It should be our goal to achieve this knowledge base without in any way undermining the future use of this genetic tool.

In closing, it is obvious that the development of CRISPR/Cas9 has undoubtedly shaped the field of biomedical sciences in a monumental manner. We now have the technology available to bring about rapid and highly specific alterations to the genome easily and thus have amassed a plethora of knowledge that will surely benefit the generations to come. However, further development and responsible usage of such avenues for knowledge rely greatly on the safe navigation of ethical matters and avoidance of any unsustainable uses that this tool may be utilized for. It is our hope that this review provides a cumulation of information that will serve informatively and innovatively, providing the needs for further development of CRISPR/Cas9 technology and its uses.

## Figures and Tables

**Table 1:** Application of CRISPR as a therapeutic tool for common monogenetic disorders of humans.

Disease	Manipulated gene	SgRNA target	Cell type	Species	*In vivo/in vitro*	Delivery	Outcome	Ref
Cystic fibrosis	CFTR locus F508 del	*CFTR* exon 11 or intron 11	Cultured intestinal stem cells from organoids isolated from CF patients	Human	*In vitro*	Cas9, sgRNA plasmid transfection	Successful and rescued CFTR protein	[28]
	F508	*CFTR* exon 10.	Induced pluripotent stem cells (iPSCs)	Human	*In vitro*	PiggyBac transposase nucleofection	Normal CFTR expression on differentiation	[29]

Sickle cell anemia	*β*-Globin	Exon 1 of the human *β*-globin gene	Blood-derived CD34^+^ cells CD34^+^ stem and progenitor cells from SCA patients	Human	*In vitro*	Lenti-viral vector	Successful 18% gene modification in in vitro cells. Wild type H b cells observed	[30]
	*β*-Globin		CD34^+^ cells	Human	*In vitro*	Nucleofection	30% HDR in CD34^+^ with 80% of them being viable and producing *β*-globin	[31]
	Glu6Val mutant gene		Stem and progenitor cells	Human	*In vitro*	Adeno-associated viral vector	Successful rectification achieved	[32]

Thalassemia	IV52-645	Gene targeting intron 2 mutation site near IVS2-654 C > T mutation	Thalassemia patient-derived iPSCs	Human	*In vitro*	PiggyBac transposon donor	Mutation corrected in the relevant site	[33]
	HBB gene CD 41/42 mutation		*β*-Thal iPSCs	Human	*In vitro*	Lenti-CRISPR V2 vector.	Cells have exhibited normal karyotype and have retained full pluripotency	[34]
	*HBB* mutation	TLTT deletion between 41^st^ and 42^nd^ amino acid producing Hbb gene	Naïve iPSCs obtained from urinary cells of *β*-thalassemic patient	Human	*In vitro*	pX330 vector	Exhibited normal karyotype and retained pluripotency	[35]
	*HBB* mutations	Exon 2	iPSCs from thalassemia patients	Human	*In vitro*	PiggyBac transposon vector	Full pluripotency or normal karyotypes and no off-target effects	[36]
	*HBB* mutations	2^nd^ intron of the Hbb gene.	Induced pluripotent stem cells (iPSCs)	Human	*In vitro*	B003 plasmid vector transfection	Normal cell types with no off-target effects	[37]

Huntington's chorea disorder	HTT gene	Promoter region, transcription start site, and expanded CAG mutation	Primary fibroblast cells	Human	*In vitro*	CRISPR/Cas9 vector transfection	Rectification of mutation successful	[38]
	Mutant HTT locus	HTT exon 1 deletion	Fibroblasts and in vivo HD transgenic mice	Human and mouse	*In vitro*	Plasmid pX330	Rectification of mutant HTT locus	[39]
	mHTT	CAG repeats in exon 1 of the human HTT gene	HEK 239 cell line	Mouse	*In vivo*	Adeno-associated virus vector	Reduction in expression of mHTT in the striatum of model mice	[40]

Duchenne muscular dystrophy	Dystrophin gene	Exon 45–55 with introduced shifts within exons	Myoblast cells	Human	*In vitro*	hCas9-T2A vector	Single large deletions with corrections in 62% DMD	[41]
	Dystrophin gene	Exon knocking	Patient-derived iPSCs	Human	*In vitro*	Nuclease-expressing plasmids	Replacing dystrophin protein when differentiated into skeletal muscle	[42]

Hemophilia	F8 gene	Introns 1 and 22 of the F8 gene	Patient-derived iPSCs	Human	*In vitro*	Cas9- and gRNA-encoding plasmids vector	Reversal of inversion back to WT situation	[43]
	Y371D in the human F9 gene			Mouse	*In vivo*	Adenoviral vector transfection	Mutation rectified	[44]

Chronic granulomatous disease	CYBB gene		iPSCs derived from phagocytes from CGD patients	Human	*In vitro*	CRISPR-cas9 vectors	Restoration of oxidative capacity	[45]
	Gp91^phox^		Patient-derived blood stem cells	Human	*In vitro*	Cas9 plasmid vector	Stable expression of gene following rectification and engraftment into mouse models	[46]

**Table 2:** Application of CRISPR as a therapeutic tool for common multifactorial diseases of humans.

Disease	Manipulated gene	Target	Cell type	Species	I*n vitro/in vivo*	Delivery	Outcome	Ref
Cancer	HPV16 genome	Exon 7	HPV positive SiHa and Caski cells, HPV negative C33 A HEK 293	Human	*In vitro*	Luciferase reporter pSSA Rep3-1 plasmid	Apoptosis and growth inhibition of cells. No inhibition and apoptosis. Downregulation of E7 protein and upregulation of tumor suppression protein pRb.	[65]
	Pten gene and p53 gene simultaneously		Liver cells	Mouse	*In vivo*	Hydrodynamic injection to deliver of Cas9 and sgRNAs	Liver tumors similar to those caused by Cre-loxP mediated deletion of p53.	[66]
	Pten gene only		Liver cells	Mouse	*In vivo*		Akt phosphorylation and lipid accumulation phenocopying gene deletion using Cre-loxp deletion.	[66]
	AsLX1 homozygous mutation	Genomic region overlapping AsLX1 mutations observed in KBM5 cells	KBM5 cell line	Mouse	*In vivo*	pX458 vector transfection	Longer cell survival was observed when compared to cells that were not rectified. Normal cell function rectified and downregulated polycomb repressive complex 2 genes. Longer survival in mice with X enograft of corrected cell lines as opposed to these X enografted with uncorrected cell lines. Correction of driver mutations in leukemia cells increases survival in in vivo mice.	[67]
	CDK 11	4^th^ coding exon of CDK11	KHOS and U 205 osteosarcoma cell lines	Human	*In vitro*	U6gRNA-cas9-2A-GFP	Decreased viability and proliferation of osteosarcoma cells. Induced apoptosis in KHOS and U20 cell lines. Reduced invasion and migration of cells.	[68]
	MCL 1		Human Burkitt's lymphoma cells. Burkitt's lymphoma xenograft models.	Mouse	*In vivo*	Dual lentiviral vector system	Apoptosis of lymphoma cells at high frequency. Tumor regression or impaired growth.	[69]
	SGCBP1		MCF-7 and MDA-MB-231 cell lines.	Human	*In vitro*	Lenti CRISPR/CAS9 vector	Inhibited proliferation of breast cancer cells.	[70]
	KLHDC4	Targeting exon 5 of KLHDC4 gene	Nasopharyngeal carcinoma cells	Mouse	*In vivo*	pX330 transfection vector	Inhibited growth, migration, cell proliferation, migration of cells, and increased apoptosis.	[71]

Diabetes	INS gene	Exon 2 and exon 3	Porcine primary fibroblast cells	Piglet	*In vivo*	px458 vector	Successful models generated for study.	[72]
	Letine/leptine receptor genes	Exon 2	C57BL/6J embryo (mice)	Mouse	*In vivo*	Microinjection of Cas9 mRNA and sgRNAs	Phenotypically identical to mice models involving the use of obese and diabetic mice.	[73]
	Hepatocyte nuclear factor 1B		Human iPSCs	Human	*In vitro*	Plasmid vectors	It provides extensive insight into the influence that HNF1B knockout mutations can have on the development of diabetes and the molecular mechanisms involved with pancreatic development.	[74]

Cardiovascular diseases	LMNA		1-cell stage zebrafish embryo	Zebrafish	*In vivo*	Microinjection	Models for the study of early-onset CCD.	[75]
	PCSK9 gene	Exon 1 and exon 2 of the PCSK9 gene	Hepatocytes	Mouse	*In vivo*	Adenoviral delivery	50% of mice showed loss of function and reduction of LDL levels.	[76]
	ANGPTL3			Mouse	*In vivo*	Adenoviral vectors	Reduced risk of CHD, reduced blood triglycerides and LDL.	[77]
	ApoE and LDLR gene	Exon 2 of ApoE and LDLR gene		Pig	*In vivo*	pGL3-U6-gRNA-PGK-puromycin and Cas9 expressing plasmid	Successful generation of pig models.	[78]
